# “Why do i have to get naked to have a sandwich made?”: The social problem of aging and cultural shift needed to facilitate aging in place

**DOI:** 10.1186/s12877-025-06199-8

**Published:** 2025-09-26

**Authors:** Danielle Jacobson, Tashani Parker, Junhee Baek, Lauren Cadel, Walter P. Wodchis, Elizabeth Mansfield, Laura Rosella, Marissa Bird, Kerry Kuluski

**Affiliations:** 1 Institute for Better Health, Trillium Health Partners, 2085 Hurontario St, Mississauga, ON L5A 4G1 Canada; 2 Institute of Health Policy, Management, and Evaluation, University of Toronto, 155 College St 4th Floor, Toronto, ON M5T 3M6 Canada; 3 Dalla Lana School of Public Health, University of Toronto, 155 College St 5th Floor, Toronto, ON M5T 3M7 Canada; 4 Leslie Dan Faculty of Pharmacy, University of Toronto, 144 College St, Toronto, ON M5S 3M2 Canada; 5 Department of Occupational Science and Occupational Therapy, University of Toronto, 500 University Ave, Toronto, ON M5G 1V7 Canada; 6 Institute for Clinical Evaluative Sciences (ICES), University of Toronto, 155 College St Suite 424, Toronto, ON M5T 3M6 Canada; 7 Centre for AI Research and Education in Medicine, Department of Laboratory Medicine and Pathobiology, Temerty Faculty of Medicine, University of Toronto, 1 King’s College Circle 6th Floor, Toronto, ON M5S 1A8 Canada

**Keywords:** Aging, Aging in place, Aging at home, Patient- and family-centred care, Cultural shift

## Abstract

**Background:**

Over the next 2 decades, the population of older adults (65 years of age and up) in Canada is anticipated to rise significantly, with the most rapidly increasing subset being racialized minorities. Many older adults prefer to age at home and in the community despite lacking services to support this goal. Considering older adults’ growth rate and preferences, the research team sought to investigate what resources were needed to facilitate aging in place.

**Methods:**

This study took place in Peel Region (Ontario, Canada), a particularly diverse region in Canada. 15 focus groups and 7 1-on-1 interviews were conducted with 42 participants (14 older adults, 10 caregivers, 18 healthcare providers). Each session included 2 parts: (1) co-designing a persona, and (2) co-designing a care package for the persona based on what they would need to age at home. A collaborative approach was taken to Braun and Clarke’s inductive thematic analysis.

**Findings:**

Overall, participants pointed to the social problem and medicalization of aging and discussed a broader cultural shift necessary to facilitate aging in place. This cultural shift was multifaceted and involved reducing fear-based health education rooted in liability concerns, caregivers and families accepting risks, a better balance between governmental responsibility and community sustainability, and looking to other optimal models of care (e.g., palliative care model, family planning model for advanced care planning). Important to shifting the culture of care was also the need to better balance standardized and personalized care. Older adults and caregivers needed opportunity to voice their needs. When older adults did not receive flexible accommodations for their specific circumstances, there were often spillover costs. Finally, important to centering older adults and caregivers in person-centred care provision was a needed shift in the consistency and reliability of care services that supported older adults to age in place.

**Conclusions:**

Participants described receiving care that was often not person- and family-centered despite this being a purported healthcare system value in Ontario, Canada. A cultural shift is thus needed in medical and social expectations of what it means to care for older adults.

## Introduction

Over the next 2 decades, Canada’s population of older adults (65 years of age and older) is estimated to rise by 68% [1], making them “1 of the fastest-growing age groups” whose long-term health challenges must be understood and addressed at a governmental level [2, p. 1]. Older adults often prefer to age in place (i.e., at home or in the community) rather than being admitted to residential care facilities such as long-term care homes [3–9]. Interestingly, 1 in 5 older adults in Canada who transitioned to residential care are reported to have needs that were analogous to those cared for in the community and thus may have been able to age in place with support [10, 11]. However, due to a lack of access to community-based support [7, 12] and scant caregiver resources [13], the ability to age in place is often limited.

Important to considering current community-based supports for those aging at home is the demographics of older adults in Canada; the most rapidly increasing subset being racialized minorities [14]. As Laher [15] aptly put it, “the Canadian population is not only growing older, but the ethno-cultural diversity of Canada is increasing” [p. 1]. Calls for home care services have thus been made to better reflect the increasing diversity of older adults in Canada [15], including a keener focus on person- and family-centred care [8]. While there is scant research on whether home care services meet the needs of culturally and linguistically diverse older adults, evidence on residential care facilities indicate that this population experience reduced social engagement, and lack psychosocial support and communication, resulting in worsened health outcomes such as delay in diagnosis or treatment [16, 17] and unfulfilled psycho-social needs [18–20].

Given the aging demographics and scant literature on the needs of culturally and linguistically diverse older adults, it is pertinent to better understand how to reallocate health and social services to support diverse older adults aging in place. The research team therefore sought to work with the local community of patients, caregivers, and healthcare providers through community engagement and co-design to answer the following questions: (1) What services and resources do older adults need and prefer to age in place? and (2) What are the barriers and facilitators to providing sufficient care to facilitate aging at home?

## Methods

### Conceptual framework and positionality

This study was guided by a critical, social-justice paradigm; underlying values influenced all stages of the research process [21]. Inherent in this paradigm is a view of knowledge as value-mediated and power relations as influencing experiences with an emphasis on being reflexive about positionality [22]. The authors utilized the “balance of care” framework, which originated in the United Kingdom and aims to understand how to reallocate resources to support older adults to age in place [23–29], especially related to those on waitlists for long-term care homes [30] and those living in rural areas [27, 31]. The authors aimed to build on the approach by soliciting rich experiential data from diverse community members to outline community needs and build equity-focused care-packages designed to support diverse older adults to age in place.

The research team comprised individuals with varying lived experiences in supporting older adults to age in place. Some team members had been a primary caregiver for a loved one aging in place, others were secondary or tertiary, and others had clinical experience. Team members were thus motivated to centre older adults, caregivers, and families in the work. The research team had varying professional experience, including senior scientists, associate professors, research associates, clinicians, and some in training. The team was multidisciplinary with expertise in public health, social work, machine learning, psychology, and specializing in topics including aging and long-term care, women’s health, and qualitative research. Researchers came from a range of religious, racial, cultural, and ethnic backgrounds and discussed how their social locations had an impact on their worldview and, therefore, on the research [32–34].

### Study setting

This study took place in the Peel Region (Ontario), a particularly diverse region in Canada [35]. Approximately half of Peel’s population are immigrants and half report low income [36]. Over half of Peel’s racialized community identify as South Asian, with others identifying as Chinese, Black, Filipino, Latin American, Arab, South-East Asian, and West Asian [37]. The 2021 census indicated that nearly 15% of Peel Region’s population was 65 years of age and older [38]. From 2016 to 2021, Peel Region’s aging population increased by 20%, representing the most rapidly rising age group [39].

### Recruitment and consent

Recruitment took place from September 2023 to February 2024. Purposive recruitment involved building relationships with diverse community organizations (spanning a range of health and social sectors, across professions) who helped engage participants reflective of Mississauga’s diverse population. The study team engaged interpreters for the translation of study materials and interviews. While not a participation requirement, the research team emphasized to community partners the goal to include diverse recipients of community care and those on the waitlist for long-term care. Community organizations and study collaborators disseminated recruitment materials. The researchers’ networks were leveraged. Participants interested in the study reached out to the research team. The option was available to submit consent electronically (i.e., signing the consent form) or verbally (verbal consents were audio recorded). Participants provided ongoing consent and were encouraged to ask questions throughout the study. Participants were informed of their right to withdraw at anytime and without consequence; none withdrew.

### Participants

Forty two participants engaged in the study including 14 older adults (65 years of age and up), 10 caregivers, and 18 healthcare providers, though many had overlapping roles. For example, some who identified as caregivers in the study were also older adults. Similarly, some who entered the study identifying as older adults and healthcare providers also described playing a caregiving role. Caregivers described engaging in care for parents, spouses, siblings, aunts or uncles, in-laws, neighbours, and friends. Healthcare provider roles included: nurse, home support worker (HSW), personal support worker (PSW), physiotherapist (PT), occupational therapist (OT), physical therapist assistant (PTA), occupational therapist assistant (OTA), medical doctor, social worker (SW), recreation therapist. To be included in the study, participants had to be an older adult or a caregiver or provider to an older adult, and able to provide informed consent. Individuals with cognitive impairment were welcomed to participate with a substitute decision-maker. No participants had a known cognitive impairment, though some caregivers and healthcare providers cared for individuals with cognitive impairment. English speaking was not a requirement for participation. 1 focus group with 2 participants was translated. See Table 1 for a summary of participants' socio-demographic information.

**Table 1:** Participant socio-demographics (patients, caregivers, healthcare providers)

Age	Sex	Gender Identity	Racial or Ethnic Identity
30–39: 6	Male: 4	Man: 5	Asian mixed heritage: 1
40–49: 8	Female: 38	Woman: 37	South Asian: 4
50–59: 7			Central Asian: 1
60–69: 9			East Asian: 1
70–79: 11			Chinese: 2
80–89: 1			Middle Eastern: 5
			“Mix”: 2
			Bi-racial: 1
			White: 9
			Latin American: 1
			South American/West Indian: 1
			West Indian: 1
			Black: 9
			Caribbean: 2
			Jamaican: 1
			Southern European: 1

### Focus groups and interviews: Creating personas and care-packages via co-design and flexible participation

This qualitative study was part of a larger sequential mixed-methods research study. The quantitative portion (reported separately with its own ethical provisions) used administrative health data (Resident Assessment Instrument- Home Care data) to divide the population of Mississauga waiting for long-term care by 5 MAPLe levels (Method for Assigning Priority to people needing community- or facility-based-services) [40] to better understand the characteristics and needs of older adults who wished to age at home. The first MAPLe level represented the lowest care needs, while the fifth level encompassed the highest [40]. The qualitative study team drew on characteristics derived from the quantitative analysis to create 5 initial risk profiles (summaries of the characteristics), which corresponded to each of the 5 MAPLe levels. The 5 risk profiles were then iteratively co-designed into more detailed personas (contextualized descriptions depicting an individual and what they might experience, including their strengths, level of independence, health and social needs; see Table 2: Persona example) during focus groups and interviews by participants who enriched the data with experiential details to better reflect diverse communities within Peel Region. Personas are known to facilitate participants’ engagement when co-designing healthcare interventions and innovations [41, 42]. Co-design is a method to engage stakeholders to work collaboratively to improve a procedure or product [43, 44].

**Table 2:** Persona example

MAPLe Level	Persona Description
**4**	Khalid is an 88-year old man. He speaks Arabic and is in touch with his faith. He lives in a house in a low income neighborhood with his wife, Layla, who is his caregiver. Layla is older and experiencing frailty and stress in her caregiving role. She is ignoring her own ailments and needs support. Khalid and Layla are finding it difficult to manage on their own. Layla has noticed Khalid showing signs of short term memory problems which impacts his ability to make decisions and make himself understood. Khalid can recognize different times of the day in his usual environment and familiar people. He uses the phone independently but needs hearing aids, which he sometimes forgets to use. He mostly independently feeds himself but sometimes has difficulty. He uses the toilet independently (though with difficulty due to limited mobility in his joints) and needs cueing for personal hygiene. He also requires help with dressing, managing medications (requires blisterpacks/reminders), and bathing (e.g., washing his lower body; but actively participates). He walks with difficulty and sometimes struggles to get in and out of bed/chair and has had a fall in the last month. Khalid now requires assessment for a gait aid. He now uses non-emergency ambulance transport for appointments (on a stretcher). He relies on others to prepare and heat meals, grocery shop, complete housework, and manage finances. Khalid prefers Arabic speaking providers familiar with his cultural practices. Sometimes he feels lonely. He is able to see his family doctor when needed at home. His children provide emotional support, however it can be hard for them to get to his house in a timely manner.

Fifteen focus groups and 7 1-on-1 interviews were conducted using Zoom software [45]. For anyone unable to attend a focus group or who preferred an individual discussion, interviews were offered. Participants were told that interviews and focus–groups could be facilitated via Zoom (or using a telephone to call into the Zoom meeting) or in-person. All participants opted for the Zoom option. Some participants chose to call into the Zoom meeting via telephone. Technological support was offered to all participants; DJ provided further instruction to 4 participants regarding how to call into or join Zoom using a computer or smart phone. When participants who had booked an interview or focus group did not show up on time, 1 of the 2 facilitators called them via telephone to provide real-time assistance to join the meeting.

Each focus group or interview focused on 1 of the 5 personas and had 2 parts: (1) building on the persona (participants were asked what resonated with them and what was missing), and (2) co-designing a care package for the persona (participants were asked about the strengths and challenges this person might experience and the necessary services to age at home). While the research team did not explicitly inquire into participants’ MAPLe level nor experience with others’ MAPLe level, DJ and TP did engage in discussion about which MAPLe level session may be most appropriate for each participant and considered this in conjunction with prioritising equal opportunity for all five personas to be explored. For example, efforts were made to assign a palliative care physician to a MAPLe level 5 focus group (highest level of complexity). Further, the time taken to build relationships with diverse community organizations allowed DJ and TP to gain familiarity with many of the prospective participants; efforts were made to assign those with experience with lower care needs to lower MAPLe level interviews or focus groups and those with higher care needs to sessions focusing on a higher MAPLe level.

Focus groups were conducted by 2 researchers; the first facilitated and supported the conversation and a second supported participant feedback and notetaking. The facilitator who engaged in notetaking shared their screen so participants could view the developing work and provide feedback. Co-designed personas and care-packages were refined after each session and then presented again at additional focus groups and interviews for more feedback. The 5 personas and service care-packages are reported in detail in another manuscript, part of the overarching project, which draws focus in more depth to the methods of the study [46].

The questions which guided focus groups and interviews were developed for this study (see Table 3: Persona development worksheet). The persona development worksheet not only acted as a semi-structured guide for the focus groups and interviews but also allowed the researchers and participants to build on the persona (by adding more contextual details inspired by the prompts). Adding these contextual details provided a bridge into co-creating a related care package, using the same worksheet, which considered financial needs, formal and informal support available, accessibility to healthcare providers, and other related social determinants of health. For example, discussion of the persona’s ability to engage in activities of daily living (ADLs) and instrumental activities of daily living (iADLs) prompted exploration into which providers were necessary to provide specific supports. After each interview and focus group, the persona story and persona development worksheet were updated to reflect the most up to date co-designed information. Each additional interview and focus group then built on this information.

**Table 3:** Persona development worksheet (focus group and interview guide)

My name is:	I am *___* years old I identify as: Man ☐ Woman ☐ Non-Binary ☐ Transgender ☐ Gender Fluid ☐
Marital Status:	
I was born in___[country]	
I have enough money to meet my daily living needs: ☐ Yes ☐ No Additional content as needed about financial needs:	
I have someone I can count on when I need something (such as a friend or family member): ☐ Yes ☐ No	
This person provides (how many hours): ☐ Per week ☐ Per day	
This person lives in the same city as me: ☐ Yes ☐ No	
Living arrangements: ☐ On own ☐ With others	
Description of current residence (housing, if applicable) and neighborhood:	
Is faith (religion, beliefs) important to you? ☐ Yes ☐ No Additional content as needed about the influence of faith and regular practices:	
Do you engage in specific cultural practices? ☐ Yes ☐ No Add additional content as needed about cultural influence and practices:	
How do you categorize your ability to make decisions about your daily care needs? ☐ Independent ☐ Needing significant help ☐ Needing supervision ☐ Dependent on someone else ☐ Needing some help	
Description of ability to do activities of daily living (eating, bathing, toileting, etc.):	
Description of ability to do instrumental activities of daily living (managing medication(s), managing finances, making meals, housekeeping, paying bills, driving to appointments):	
I currently interact with the following types of care providers:	
I have a Family Doctor I can visit when I need medical attention ☐ Yes ☐ No Additional content as needed about family doctor:	
I prefer to receive services in my primary language ☐ Yes ☐ No	
Other important details about me:	
The types of things I need support with are:	

Co-design of both the persona stories and related care-packages (see Table 4: Care package for MAPLe level 4 persona) informed the findings reported in this study; these were interrelated tasks. The discussion of the details of the persona story informed what was viewed as needed in the care-package for that persona. For example, discussion of the persona’s ability to engage in iADLs led to conversation around advanced care planning, the related support needed, who might provide that support, and the time commitment involved (care-package).

**Table 4:** Care package for maple level 4 persona (Khalid)

Provider	Service	Hours
Caregiver	Assists with translation, meals, feeding, laundry, nighttime bathroom use, shopping, cooking, caring, laundry, finances/pay bills, socialization, advocating. Barriers: Unmeasured impact of caregiving on caregivers (time off work, lost economic value, mental health implications), need flexibility to self-identify individual needs, need more caregiving services for those without family, need education about managing medical conditions, lack of respite.	5 h/day
Dietician	Assess individual food needs and provide related resources. Barriers: Need culturally relevant recommendations and increased understanding of the nutritional value of cultural foods.	Not specified
Personal Support Worker (PSW)	Assists with dressing, bathing, laundry, taking out garbage, housekeeping, shopping, cleaning fridge, preparing meals (culturally appropriate food), cueing/giving medications, carrying out exercise, getting ready to go out, preparing for sleep at night. Barriers: Need culturally appropriate staff who speak the older adult’s primary language, more flexibility needed for older adults and caregivers to decide what to do with designated hours, need a consistent provider, PSWs sometimes do not show up, PSWs sometimes only show for 15 min despite 1 h being scheduled, limited scope of work (e.g., PSWs often do not assist with iADLs), older adults require a more gentle touch than often provided, lack of professional incentives for PSWs, need more frequent checkpoints to ensure safety, accountability, and oversight to protect older adults.	−2x/day (morning and night)
Occupational Therapist (OT)	Initial assessment, assess home entrance/exist accessibility (e.g., ramp, transport chair), recommend equipment/assistive devices (and assess function and use once installed), provide resources for coverage of related equipment, provide follow up when change is needed. Barriers: Not enough coverage for equipment/assistive devices.	−2-4x/month (initial) −1x/year (follow-up)
Physical Therapist (PT)	Assess mobility and gait, provide relevant exercises and how to incorporate these into everyday life, focus on fall prevention. Barrier: Not enough follow up to practice exercises together.	−2x/week (10 weeks)
Social Worker (SW)	Needs assessment, support in finding and applying to funding opportunities, provide knowledge of community resources, weekly mental health checks (for older adult and caregiver), discuss end of life planning and power of attorney, counselling. Barrier: Need more flexibility.	−1x/week (10–15 min)
Care Coordinator	Assess needs in person (at the older adults’ home), ensure that services (or a plan) are in place to complete daily tasks (e.g., garbage, laundry, food, grooming, etc.), oversee care providers to ensure older adults’ needs are met. Barriers: Not specified.	-Ongoing
Family Doctor	Home visits for care, follow up calls and monitoring, renewing prescriptions, in-home vaccinations, assessment/prevention of cognitive impairment (1x/year). Barriers: Gap for home visits, requires a stronger clinical acumen for older adults, need for services in the older adults’ primary language.	1x/1–3 months
Geriatrician	Follows active medical issues, introduces new supports, mobility assessment, assess if main-floor set up is required (bedroom, bathroom, and caregiver on the main floor), prescribes medicines, inquires if the caregiver is managing. Barriers: Gap for home visits, needs to provide services in the older adults’ primary language.	1x/4–6 months
Nurse	Part of geriatric outreach team to do home visits. Barriers: Not specified.	-Not specified
Pharmacist	Delivers/monitors medications (and interactions; especially when medications are prescribed by different doctors), prepares blister packs, provides education about medications in person (including information about renewals and new medicines). Barriers: Need for provision of a list of current medicines (e.g., to take to the emergency room).	-Not specified
Home Support Worker (HSW)	General cleaning of the whole house (emphasis on bathroom). Barriers: Lack of funding.	−1x/week
Lawyer	Provides advice about assets, provides tips to avoid being taken advantage of, assists in putting a power of attorney in place. Barriers: Not specified.	-Not specified
Paramedic	Well-visit checks. Barriers: Not specified.	-Not specified
Recreation Therapist	Implements fall prevention programs, provide encouragement to take care of oneself, work to prevent unnecessary hospitalization through exercise and cognitive activities. Barriers: Needs to be covered under home care services (this service is currently fee for service).	−2x/week (6 weeks)
Respite	Provide relief for the caregiver (e.g., time for them to prioritize their own wellness), assist with housekeeping and groceries, connect older adults and caregivers to relevant program/services. Barriers: Lack of funding to support access to this service, lack of day programs which may be a source of respite (including transportation).	Not specified
Religious Institutions	Provide in-person and online services in the older adults’ primary language, provides in-person community and social activities, offer support with housing, sending volunteers to provide assistance (e.g., with shopping, meals, etc.). Barriers: May be difficult to connect to online services independently.	-Not specified

Each interview involved 1 participant while each focus group included between 2 and 4 participants. To create an open and safe space for in-depth discussion, 2 types of focus groups were created: patients/caregivers participated together and providers participated together (see Table 5 for the number of sessions conducted for each persona). Participants in both the interviews and focus groups had the opportunity to engage in multiple interviews and/or focus groups across the project to facilitate continual learning, co-designing, comfort and opportunity for contribution to building the personas and care-packages [47]. 6 participants who had previously participated in a focus group engaged in a second focus group, 3 in a third, and 2 in a fourth. The authors aimed to have an even spread of participants (including participant types) across each of the personas. Participants indicated appreciation for the collaborative nature of the sessions and expressed benefitting from shared information from co-participants about available resources in the community when co-building the care-packages. Participants were compensated for each session with a $50 CAD gift card to a place of their choosing.

**Table 5:** Number of participant sessions conducted for each persona

	Older adult/caregiver sessions	Healthcare provider sessions
MAPLe Level 1 Persona	2	2
MAPLe Level 2 Persona	2	2
MAPLe Level 3 Persona	2	3
MAPLe Level 4 Persona	2	3
MAPLe Level 5 Persona	2	2

### Data analysis

All focus groups and interviews were audio recorded and transcribed verbatim. Identifying information was removed from transcripts and pseudonyms were assigned. Transcripts were checked against the audio recordings for accuracy. Using NVivo software [48], a collaborative approach was taken to inductive, codebook thematic analysis [49–51]. The first 3 transcripts were independently coded by DJ, JB, and TP, who then came together to review the codes. The study parameters dictated the informational need to better understand the services required to facilitate aging in place, however, the research team did not use a fixed codebook. Rather, the codes evolved as new understanding of the data emerged and as the team further reflexively engaged about their impact on analysis [50]. Therefore, at times, this approach took on qualities of reflexive thematic analysis (e.g., in the authors’ consideration of the researchers’ subjective impact on the research process and data analysis). DJ continued to independently code the remaining transcripts. JB double checked codes for half of the remaining transcripts and TP reviewed codes for the other half. Convergences were plentiful with few divergences. DJ then amalgamated codes with similar meanings and eliminated codes that were no longer relevant. DJ and TP discussed emerging themes, what participants indicated was important to them, and the nuances captured within the themes. Final themes were refined and subthemes generated (these were verified with the larger research team) prior to beginning analytic writing. The authors reached out to community organizations whose members engaged in the study to present findings; the results resonated with participants who expressed continuing to experience the challenges identified in this research.

## Findings

### Theme #1: Shifting culture of care within families, health system resources, and models of care

#### Subtheme #1: Cultural shift in caring attitudes to accept the risk of supporting an older adult at home

Participants described a three-pronged cultural shift necessary to support older adults aging in place: (1) shifting from risk avoidance to acceptance of the risks of aging in place; (2) shifting from fear-based education to increased information on “what will make it [aging in place] work”, (3) shifting from a “hands off” caregiving culture to one that is more “hands-on”. In this context, a cultural shift refers to a change in dominant norms and practices in older adult care.

Those who supported older adults aging at home, particularly those without cognitive impairment, had to accept an element of risk; caregivers and families were often fearful of falls or negative outcomes. Maria (palliative care physician) said:

You would need a cultural shift to say “I accept the risks of this person being at home. It’s okay if they fall. It’s okay if they’re home alone for a little bit.’ … There’s no way to mitigate the risk. She could fall any time. She could forget to eat anytime. There’s not really a solution … unless you accept a little bit of that risk.

Clara (social worker) clarified that this risk extended to any care environment: “They fall in hospital. They fall in long-term care. You can’t bubble wrap people”. Fear was often rooted in healthcare interactions, particularly in the approach to education on caring for an older adult, which was meant to cover liability. Clara (social worker) continued:

We provide a lot of fearful health education from the hospital … to cover all our liabilities. We cover all the risks and you’ve scared this poor caregiver now who’s already overwhelmed. … I get calls in, “am I going to be liable? Because my mom wants to be at home. And I know I can’t cover 24/7.” … We also create that fear.

She often heard from caregivers that there was a lack of focus on what would make aging in place “work”: “[They say], ‘you’re not giving me a lot of help and a lot of tangible information, like little practical creative ideas of how to make this work’”.

Another part of the cultural shift to support older adults to age at home was countering an often hands-off caregiving culture (i.e., with limited direct caregiver involvement). Maria (palliative care physician) elucidated: “[Caregivers] won’t accept the education on caregiving unless you change the culture of it being an expectation that they do the caregiving. It’s hands off. It’s like, ‘I can’t wipe his bum. … That’s just not something I can do’”. She continued to describe the challenge when some families were not emotionally prepared to assume the caregiving burden:

I had a family the other Day. She had dementia for 5 years. They had been clearly told about the progression. … But the daughter was angry: “What do you mean, she can’t just stay here [hospital]? I don’t want her to go to long-term care. I don’t want to take her home.” … They should have been able to take care of her at home. They just emotionally couldn’t wrap their head around it.

Although participants described some caregivers and families who “will just draw a line and be like, ‘I can’t [engage in hands-on care]’”, Priya (palliative care physician) highlighted that there were many who “self-sacrificed to the nth degree”, who say, “I might die too when this is over. But I’m going to help as long as I can”.

#### Subtheme #2: The need to better balance the responsibility of the healthcare system with caregiver and community sustainability

Another facet of the shift necessary to facilitate aging in place was better balancing government responsibility (e.g., providing resources for older adults aging in place) with community sustainability (e.g., building caregiving capacity). Maeve (OT, occupational therapist) described the difference between building community capacity for older adult care and abdicating the national duty to provide support:

It’s about community development and capacity building in a way that doesn’t make people … feel like, “oh, the government’s trying to get out of paying for things”, dumping everything on the community, everything on the family. … There’s a huge difference between delegating responsibility, building capacity, and abdicating responsibility. Unfortunately, we’re in a political climate where there’s a lot of abdication of responsibility or at least pushing things off to the private sector and to individuals.

Government abdication of responsibility manifested as a lack of resources for older adults, even when willing caregivers and families were in place. Amal (PT, physical therapist) described,

I know exactly what this person needs. Needs a good house or apartment to live, needs the right food … but none of them are available. And the wait times for all these resources are months and years. … The biggest problem is we don’t have the resources.

Resources were only available reactively (e.g., to someone experiencing active disability) rather than proactively (e.g., to someone with more independence who, with resources, may prevent the progression of ill health).

At times, some caregivers and families had unrealistic expectations of the government. Jayden (nurse) said: “The belief in the community is that nursing is going to come in the home 24-hours a day and take care of the ageing population, which is not true at all. There’s minimal supports that are out there.” An insufficient balance between system responsibility and community sustainability led some older adults to feel like they were a burden. Luis (older adult) described:

If anybody wants to end their life, there should be a way to do it [laughs] … People are really frustrated with the life especially in old age when an individual is very lonely and not getting any support needed. PSW [personal support worker] or government support is very limited and some people I know, they are just frustrated. And there should be a way that instead of giving trouble to the community … there should be a way to let it go.

Ming (older adult) explained how having unmet care needs was “a terrible feeling but we have to start to accept life as it is and do the best we can”.

#### Subtheme #3: The palliative care model as an exemplar for shifting care culture

Palliative care physician participants described the palliative model as well suited to optimize care for older adults. Priya (palliative care physician) conveyed the palliative model as “good care”: “People … want palliative care because they want these things [services]. But I would argue that’s not palliative care. It’s just good care”. Maria (palliative care physician) described the 3 pronged palliative approach, which would benefit older adults’ care: (1) planning for and education about symptoms, (2) comprehensive assessment and response to changing needs, (3) end-of-life care planning:

You basically need a 3 pronged approach. You need a strategy around the really early days when they’re still well and functional around planning and education. … Where they’re starting to have symptoms, you need some way to constantly assess how they’re doing and increase their services as their needs increase. … This is the stage where we need to start advanced care planning. … Then you’re going to have the end stage.

Part of the palliative care model’s first prong was easing caregiver anxiety—a tenet also important to older adult care: “[A social worker will] sit with families and talk about some of those really just practical planning issues. And often in doing that, you’re also addressing the anxiety that people have in their caregiving role” (Priya).

Aligned with the second prong, Priya (palliative care physician) explained how having providers able to respond to changing needs in a timely manner was important:

If you put that palliative approach on to this chronic senior care, these folks who are in lieu of long-term care, I don’t think you can go wrong. … You need a team that’s sort of available on standby who can support when something’s changing. And I don’t think that’s horribly resource intense but I think it is a layer that’s missing.

She continued to describe the responsiveness of palliative providers:

In the palliative care world, before you call 911 … the nurse [or a support person] will come and see you, figure out what’s going on. These are people who have some basic healthcare information about you. They may not have met you before, but they have access to some parts of your record. … That nurse can either solve things or they’re going to call the doctor and then the doctor will help you figure out, should I go to emerge? Can we do something at home?

Maria (palliative care physician) echoed: “I have a lot of patients who are willing to say, ‘I’m good. Just keep me at home. I just want to be able to call somebody when I need help’”.

Part of having someone to call for help was a responsive family physician. Priya (palliative care physician) explained, however, that family physicians were often not accessible to older adults:

These folks are often in a family doctor void when they’re homebound. … It’s a huge problem. And so a lot of these folks, even if they want … to stay at home … they don’t have any means to do that because they don’t have anyone to call to help them.

Gabriel (geriatrician) explained that a barrier in conducting home visits for family physicians was that “they’re just not compensated properly”. He continued to explain that to do a home visit, “a family doctor will get $30” and that “you just can’t make a living doing that”; comparatively, “geriatrics and palliative care … get $500 to go and see a patient on a home visit”. For family doctors, the compensation for the work they were doing to support complex patients at home was insufficient. Because of geriatrician and palliative physicians’ limited capacity to do home visits, it was important for “older adults to be able to access their primary care doctors whether they’re homebound or not”.

#### Subtheme #4: Family planning and prevention models as inspiration for shifting advanced care planning culture

Part of the cultural shift participants discussed was better education and reconceptualization of advanced care planning, however, Trinh (older adult/caregiver) explained that this was not often prioritized: “When you’re young you never think of aging. … You think of paying the mortgage, getting the family, everything except aging. Then it hits you. … You’re on your own. … Preparing for old age is quite the task”. Ines (older adult) emphasized the importance of advanced care planning education prior to 65: “Most of these things start after 65. … Before that time comes there should be more education to allow people to know what can happen to them when they get older and to put things in place”. Camila (older adult/caregiver/nurse) suggested educating older adults via television (TV) programs: “Seniors watch a lot of TV. So why can’t we have programs like that … about advanced planning?” Clara (social worker) proposed this education beginning in high school, akin to financial literacy curriculum: “Why aren’t we integrating schools? … Train the high school students. … It’s the same as financial literacy. It’s part of your financial plan, your healthcare plan”.

Priya (palliative care physician) described how aging should be planned for “the way we plan when we have a baby” but that “we just culturally don’t do that”: “We think, ‘okay, so I’m going to have to have a kid. I’m going to need to take some time off work. How much time? … How much is that going to cost?’ … They don’t do that when people are at the end of their life”. Maria (palliative care physician) explained that reconceptualizing care planning for older adults required government resources akin to those available for child-care: “If we think about the childcare model … you get EI [employment insurance] coverage for that time to take maternity leave. … You have $10 a day daycare now. Where’s that for the elderly?” Gurinder (older adult) specified that this may include “go[ing] somewhere, they have some activities, they have a lunch, and then they ship them back home”. Akeelah (recreation therapist) expanded on the particular need to accommodate ethnic and linguistic diversity in such “home daycare” programs: “Can we do that for older adults? Where then I can cook the foods you’re familiar with, speak the language you’re familiar with?”

Camila (older adult/caregiver/nurse) highlighted support needed for community organizations that run culturally appropriate day programs:

Seniors may feel more comfortable because they’re already in their community, they know the language … the culture. … If the government really wants to support seniors staying at home, they probably should consider working with these community cultural centres … and fund them to organize these services.

Rana (OT) clarified that a more general “EI program” existed to support older adults, yet, it was not “enough for caregivers … to take on that burden” as it would “take away time from their work”.

Another part of shifting culture on advanced care planning was prevention. Trinh (older adult/caregiver) “noticed medicine is not anymore preventative. … You get the care if you are in an emergency”. Part of preventative care planning was early connection to resources. For example, Lin (caregiver) described barriers to registering with home and community care: “The first line would be the family doctor … they should automatically register [with home and community care]. … I find there’s a delay … they [doctor] wait until the situations kind of get really bad and then things start happening”. Early cognitive tests were another element of preventative care planning. Gurinder (older adult) explained that “anybody past 80 that is living alone should have a … test of any form of mild cognitive impairment”. Saoirse (older adult) echoed: “You don’t have someone around you to really recognize or see what’s happening. … It can just stay there and develop when you probably could have gotten some help earlier”.

Maeve (OT) gave institutional context on why it was difficult to weave prevention into care planning:

The healthcare system is an illness cure system. So our assessments always are focused on “what is your problem” and “let me give you the solution”. … Aging is not a disease. When people live in an ageist society with few or no resources and they are ignored, … the system in and of itself … is a big part of the challenge.

She conveyed that without preventative advanced care planning, older adults “only got connected to service through 1 of the bottlenecks of emerge [and] primary providers … medicalizing a social problem”. Maria (palliative care physician) expanded on how social and healthcare institutions needed to have better collaboration to avoid treating social problems as medical issues (medicalization) and to better engage in broader preventative planning. For example, she highlighted that immigration policies lacked healthcare consultation resulting in more people immigrating to Ontario than the healthcare system had capacity to accommodate:

The system could handle the volumes before and it just can’t now … You have a much more traumatized immigrant population. … They’re not coming in as healthy, well, high functioning immigrants, I think, as they did in the earlier times. … In the last few years as well, which it’s awful to say, but puts a huge stressor on the system. … Do not add more people to our city! We cannot handle it. … They have nowhere to be cared for! You’re not planning around this population’s healthcare needs at all. You just keep adding more.

### Theme #2: Shifting the balance of standardized and personalized care: flexibility as a facilitator to person- and family-centred care

#### Subtheme #1: Flexibility in services required to address older adults’ instrumental activities of daily living (iADL)

Part of the social problem of aging and cultural shift required to care for older adults was service flexibility. Fiona (geriatrician) explained that “[government funded] PSWs … are guided only to do hands-on personal care … basic ADL (activities of daily living) tasks … that’s not what people need to stay at home”. ADL tasks included basic physical needs (e.g., bathing, toileting); iADL tasks encompassed overall well-being (e.g., cooking, cleaning). While ADL support was necessary to age in place, it was not considered sufficient without support of iADLs. Gabriel (geriatrician) agreed that “there’s really no government service, at least in our region, that does [iADL support]” and added that “someone like Jean [persona] who has chronic medical conditions needs someone to come with her to appointments and also translate … [and] doesn’t need a PSW to come in twice a day to help with bathing and dressing”. However, he explained how “that’s the threshold for our current system. … You only will get those [iADL] supports … if you need help with those 2 things”. Lin (caregiver) said, the “whole system needs overhaul”. Gabriel expanded that this was because older adults often had to be “ill enough” to be eligible for ADL support. He proposed preventatively reallocating PSW hours to support iADLs *prior* to a health decline, to help older adults “stay independent and stay at home”.

Since PSWs could only be solicited to support ADLs, Fiona (geriatrician) described the misuse of hours to work around this rule; 1 older adult did not require assistance with a bath, yet requested one to bring in the PSW, and *then* asked for unofficial assistance with a meal:

1 of my patients who was 94 thought she was fine doing a bath but she needed help making a meal. She said, “Why do I have to get naked to have a sandwich made?” … We have a huge segment of our population … those middle MAPLe’s who’ve got … a little bit of functional decline, perhaps frailty, and they need help with their household chores.

Andrea (PSW) described how PSWs used to have more flexibility to care for older adults: “Back in the day, I remember PSWs used to do these things. We would take them grocery shopping. And what happened to that? It’s terrible”.

Trinh (older adult/caregiver) explained that many faced financial barriers to hiring private iADL support to age in place: “[Those who] don’t have as much financial resources … have to depend a lot on the government services. … You’re at the mercy of the government”. It was especially challenging to afford private services for individuals who immigrated to Ontario and were unable or did not find employment. Esha (caregiver) explained: “She [persona] don’t have no money because she never worked in this country. … And so I think they should give the caregiver some sort of assistance. … Some people are really struggling because they are really under the low income”. Kamala (palliative care physician) agreed that “the more money you have, the more likely you can stay at home. The less money you have, the less likely you can stay at home”.

Participants outlined the danger when older adults aging at home lacked access to iADL support. Luca (older adult) pointed to the risk of meal preparation:

It’s pretty dangerous if either Khalid [persona] or his wife is trying to prepare food and get the pot on the fire. A friend of mine just told me his mom put something on the stove and went away to do something and the next thing you know the [fire] alarm went off.

Camila (older adult/caregiver/nurse) highlighted that without support, key tenets of health, like nutrition, could fail: “She [persona] can cook, OK? But can she *really* cook? … My mom … can boil dumplings or instant noodles or whatever. Is that really cooking? Its not adequate nutrition. … Meals is the thing that I really see they need help with”.

#### Subtheme #2: Flexibility required to self-identify needs and receive accommodations

Older adults were not static at a single MAPLe level (level of care needs and independence) but rather fluctuated. Leeway was thus necessary to self-identify needs and obtain accommodations. James (geriatrician) pointed to the importance of “finding the right balance between standardized and personalized care”:

There still needs to be some flexibility … for the caregivers to be able to say, “actually, this is the 1 thing I’m struggling with most”. … There will also be aspects … that, yes, everyone like them … would need more PSW support … more access to family doctor.

However, Gabriel (geriatrician) said that services currently “don’t actually align with real life and what people really need”. Sunita (caregiver) conveyed the gap between standard service and what she needed: “My parents don’t need PSW … but I need help with physio. … It shouldn’t be like pulling teeth. … If you want the seniors to stay out of [long-term care] homes, give them what they need”. Sunita’s parents were eligible for but did not require PSW support, rather, they needed physiotherapy and were unable to request this beyond standard care. Fiona (geriatrician) explained: “the problem is the system is designed for the system and not for the patients”.

Caregivers’ knowledge of older adults’ needs could also inform more efficient services, saving time and money. Sunita (caregiver) detailed how she could self-identify some accessibility needs and did not require time consuming assessments:

There were 3 raised toilet seats. … Last week for some reason, it just went [broke]. So, I called the [Organization 15] … I told her specifically, “we have XYZ [assistive devices] in the home. What I just need is the toilet seat”. … I’m still waiting for someone to call back to do an assessment, which will take an hour and a half. And *then* send someone out, and *then* decide if they’re going to get it. … All this time and money going into that when you’re dealing with someone who can tell you, “okay, it’s just this”.

Trinh (caregiver) highlighted how service hours were broken up inefficiently and could be streamlined: “[Organization 15] gave him 2 [PSW] hours a Day … 1 hour in the morning, 1 hour in the afternoon. … Why is the system set up that way? … More can be done in the 2 hours than chopping it up.” Using both PSW hours at once would allow for consistent providers, increased client comfort, and reduced PSW travel time.

Another part of self-identifying needs within standard care was the ability to request “culturally specific” providers, which Binh (HSW; home support worker) explained was not possible:

Everything needs to go back to what he’s [persona] culturally comfortable with. … [If] clients aren’t comfortable with you, you can’t give the care they deserve … because there’s not that trust there. So it all comes back to that big “T” word. Trust. And in his case, that goes back to his culture.

Cultural and linguistic alignment was viewed as vital to building trust and to person- and family-centred care. Akeelah (recreation therapist) elucidated that “culturally specific doesn’t always work out … which can become problematic to developing … therapeutic relationships”. Jayden (nurse) conveyed grave outcomes when the need for particular providers was thwarted:

I had a patient … he was cognitively impaired. … He got paranoid about a male PSW. … [I] told the care-coordinator to keep the male PSW out of the home. … Another male PSW came and he [older adult] got a little bit aggressive. … Police were called, they brought him to emergency, then they admitted him to rule out some delirium, Blah, blah, blah. Somebody gave him Trazodone at nighttime. He aspirated and died. … Why would you be sending in a male PSW when I distinctly said the person needs a female?

Escalating from the original issue that individual needs were not considered in the provision of standardized care, a harrowing outcome ensued that may have otherwise been prevented.

#### Subtheme #3: Funding and service flexibility prevents system cost implications: Consequences of not having needs met

Unmet needs due to a lack of flexibility in government-funded services at times led to system cost implications. For example, James (geriatrician) described that older adults who ended up in the hospital but were unable to get into long-term care were given a temporary service package, in which increased home care was provided by the government for a limited amount of time. If the person did not get admitted to long-term care by the end of the service package, they would end up back in hospital and repeat the process, resulting in system cost implications:

As a way to get you out of hospital, we’ve implemented some kind of [program], it’s called Enhanced Integration … where you get 8 h a day [of in-home PSW support]. … The idea is that you’re on a crisis list for long-term care. … It’s only 120 days, and then all the supports stop, there’s just nothing. So, if they [the patient] didn’t make it into a long-term care home within that time, they just come back to hospital. … It’s really the turbo boost on the Mario Kart that doesn’t last forever. … And then everything kind of falls off and is a disaster.

Priya (palliative care physician) echoed the need for service resources to be flexible to prevent patient suffering and avoidable cost implications:

Some flexibility, again, in how current services are bundled and allocated that doesn’t necessarily require you to land in hospital first [is necessary] in order to adjust that service package to meet the needs of the family. … When you look at the cost of everything … it doesn’t make sense.

Funding was another element of government support that required flexibility. When needs went unmet due to a lack of funding flexibility, further system cost implications ensued. James (geriatrician) reinforced how “something really small creates a huge impact”:

[The older adult] was on high dose diuretics and so he was having to go to the washroom way too often. And he was too frail to get up and go. So, he’s wearing incontinence products. But they were too expensive and they’re not covered. So, to prevent himself from having to use so much incontinence products, he cut down on his water pill, which obviously sent him into heart failure, which he was then admitted to hospital and remained in hospital for another 3 or 4 weeks. … So, if we … covered the incontinence products, our system could have saved $250 to $300,000! Like, what!?

It is possible that preventatively funding the incontinence product itself may have relieved not only a burden of cost on the system but also patient-suffering. Binh (HSW) described that incontinence products “can be a large financial burden if they’re not [covered]” and how “a lot of clients I see, that can be where a lot of their money goes”.

Given the harms that may arise during a hospital stay, which led to both patient anguish and system cost implications, James (geriatrician) continued to explain the importance of preventative, flexible services that worked toward “hospital avoidance”:

What I see more of is, they were managing okay at home, then they have something simple, like a fall, and they come into hospital, and then they’re in emerge overnight. They are confused, so they’re agitated, so they get a bunch of sedating drugs, which then makes their confusion worse. They now get admitted for a week or more. They’re not mobilising, so they lose all of their ability. What they had is hard to get back. And then it’s this downward spiral. … What I’m talking about is hospital avoidance or admission avoidance. But again, we don’t have good pathways for that.

### Theme #3: Shifting consistency and reliability: Centering older adults and caregivers in care provision

#### Subtheme #1: Piecemeal care and the importance of a consistent care-coordinator

Maeve (OT) described the importance of an effective care-coordinator: “information is gold but people also want somebody there with them walking the walk … being their tour guide”. However, Rana (OT) explained that sometimes, families “don’t even know who their care-coordinators are”. Lin detailed the difficulty of getting in touch with a care-coordinator:

The key is the initial contact and getting connected with the system or somebody to help you navigate it. Without that people are just lost. … I went through the challenges in the beginning of trying to navigate [alone], and the tears, and the frustration.

Without a known care-coordinator, Jan (caregiver) gained knowledge independently: “Nobody is calling us to say, … ‘call this number and this is what you do’. No! We have to basically do our own research. … So that’s 1 of the biggest frustration”.

Ravi (caregiver) discussed not being remembered by providers:

It’s not easy to communicate to someone when we need it. … They make us feel like they’re not remembering us or they just want to give you something and you do it, you complete it. And they’re not … supporting us.

Kamala (palliative care physician) detailed the particular challenges of navigating healthcare system silos without a care-coordinator:

Imagine you’re a caregiver. … [OT] comes and she’s from [organization 15c] and she talks to you about what she’s going to do for you. Then you get a phone call from another agency and that’s your nurse. Then they come and leave a binder. … Then you get your PSW and they’re from [organization 15e]. Then they give you a folder. … I’m giving you a sense of how complicated this is. … You’re trying to just figure out “who do I call, and what do I do, and I’ve got 3 different binders”. … It’s such a nightmare.

Each silo had its own administrative contact and lacked sufficient coordination across sectors. Maria (palliative care-coordinator) said, “[it’s] a very, very piecemeal system”.

Farzana (older adult/caregiver) suggested giving families a print-out of different healthcare sectors, and their roles and contact information:

Could a sheet be given out at the time of the care-coordinator first meeting? … To say, “these are all the things … you might be qualified for”. So it gives me an understanding that if there’s a continence problem, or whatever the case may be, I could be on top of it and say, “hey, I know there’s something because I saw it on the sheet”.

Amal (PT) explained that “care navigation is an area that … we are behind” and proposed rethinking a holistic approach to who plays the care-coordinator role:

Care navigation is something that needs to be addressed within our community and should be covered, at least for people who cannot afford it. … My concern is that sometimes if you put like somebody who is not a regulated healthcare provider, I’m not saying they’re not doing a good job, but they have a limit. … Whoever is going to be having this role … needs to really, really look at the person as a whole.

Maeve (OT) explained the importance of having an in-depth comprehension of the community served: “We need to grill down. … You can’t be a good patient navigator if you don’t know your community. So you need to know those resources”.

#### Subtheme #2: Consistency and reliability as a facilitator of trust and recognition of needs

Despite PSWs being “the lowest paid workers in our healthcare system”, Fiona (geriatrician) explained that “they have the most important job”. However, reliability fluctuated. At times, PSWs did not show up for shifts, leaving older adults without care. Fiona continued:

[Older adult was] in the hospital for 6 weeks. … The team discharging him set up PSW. … His PSW didn’t show up. … The next day, the PSW came and found him covered in poo from head to toe in bed. Hadn’t been fed and hadn’t taken his medications.

When PSWs did show up, Rana (OT) explained it was often not a consistent provider due to workforce constraints: “Everybody’s burnt out. … Every day I think different PSWs show up. … They always request it [consistency], but it’s just never or not often implemented because of the high turnover and short staff”. Participants were empathetic to the vulnerability of letting a new person into one’s home and acknowledged that a consistent provider facilitated trust. Ayaan (OT) said, “I would be also very cautious about who’s coming into my home when I’m alone”; Navdeep (OT assistant/PT assistant) conveyed, “it’s a huge trust thing to let this person in”.

Lin (caregiver) highlighted that having inconsistent service providers was particularly harmful to older adults experiencing cognitive decline: “They want to send 3 different PSWs to care for 1 person who has Alzheimer’s. … They’re having a stranger coming to provide personal service for them and tomorrow it’s a different face. … How is that providing any sort of support, compassion, and dignity?” Akeelah (recreation therapist) agreed that inconsistent providers for older adults with cognitive decline was “1 of the more consistent negative comments” she heard from clients; without consistency and knowing the older adult, providers “don’t know the best ways to interact with them”. Having a consistent provider was important to learn about the person behind the patient and “what their triggers are” (Clara, social worker).

Another element of inconsistency was with regard to the time service providers arrived. Jan said:

I had a rude awakening this year. … I didn’t know it was that bad … [PSWs] showing up at 12 o’clock to do her breakfast. … That’s the first visit. … There were Days when only 1 person showed up at 8:30 [pm]! So what are these individuals who are living alone … supposed to do?

When PSWs did not show up on time, a caregiver had to step in. Andrea (PSW) described: “Some of them [caregivers] are going through hell. … Having to get up at 4 am just to go take care of their mother while they’re also raising their grandchildren and going to work. And it’s just brutal”. In this context, the caregiver lived separately from their parent and grandchildren and needed to travel to engage in caregiving activities. Aisha (OT) elucidated that when PSWs did not reliably show up, and caregivers could not fill in without notice, hospital readmission occurred: “Families that we previously sent home with home care [PSW support] are telling us, ‘they never came’”.

The often inconsistent, unreliable care described posed a barrier to person- and family-centred services. Gabriel (geriatrician) explained that building trust with a consistent, reliable provider was a “massive gap” that could not be overlooked due to its impact on patient-engagement, particularly for individuals with past trauma:

Because of past trauma there’s hesitancy for people like Jean [persona] to engage with the healthcare system. … It takes time … before they really feel comfortable. … That is a massive part because people won’t access the care properly, because they feel that “this person [provider] may not treat me properly and I have to be wary”. … To them it’s like, “Well, another institution, I’m not familiar with it. I don’t know how I’ll be treated”.

Luca (older adult) affirmed the concern of lacking trust in the healthcare system and explained how this was rooted in inadequate accountability and oversight of providers:

There has to be accountability. There has to be oversight. There needs to be checks and balances. So if someone is coming into my home to look after me or whatever, there should be somebody … that is going to monitor what’s happening and do check points … to make sure that things are legit. … There’s so many windows of abuse.

#### Subtheme #3: Providers with high clinical acumen and capacity to treat older adults as central to reliable, preventative care

Part of reliable, preventive care of older adults was a strong clinical acumen, which included attention to a care plan’s social considerations. Fiona (geriatrician) described a patient’s doctor overlooking barriers to obtaining their prescription on discharge, leading to hospital readmission:

[Physicians] send them home and don’t even fill their prescription. They don’t fax it to the pharmacy. They put it in their hand. What good does that do? … I just saw a guy yesterday, he was discharged on the [date] and back in [the hospital] on the [date; 3 days later]. And he said to me, “I never got my prescription filled”. … So much of supporting people at home … is a lack of common sense on the part of the healthcare providers. … They don’t think, “jeepers, I’m filling out a prescription. How he is going to fill it?”

Another part of the “common sense” integral to centring older adults in their care, was thorough investigation of symptoms. Maeve (OT) explained: “Diagnosis and prognosis is job one. If you don’t have that, stop making recommendations. … Let’s not just assume an 86-year-old woman who’s got memory loss, ‘oh, well it’s got to be aging’. … I’m on the bandwagon in terms of ageism but it’s so rampant”.

A strong clinical acumen for older adults’ care also included knowledge of medications. Akeelah (recreation therapist) explained that “once you start medical intervention it’s not always helpful. It can also be harmful. … Medications then lead to more medications which can increase his [persona’s] risk of falling”. James (geriatrician) described that “meds are probably 1 of the things that I see a lot of iatrogenic harm from”:

It’s just these continuous cascades. … This person might have been fine. … Maybe they’re getting a bit more agitated or a bit more kind of restless at home. So the wife calls the family doc, and the family doc … prescribes Ativan (lorazepam). But it’s well known that Ativan causes falls and cognitive impairment. … So as a geriatrician, I would never use it. … It has to do with the capacity of medical professionals to understand the implications of the drugs that they’re prescribing. And so, yes, this person now has a fall, breaks a hip … and comes into hospital. And I can trace it all back to, ugh, if we had just not done the Ativan.

She detailed the “flip side” of knowing which medications facilitated avoidance of challenges:

On the flip side … there’s someone who has really bad behaviours … and family doc didn’t feel comfortable prescribing anything. … They came into hospital and the family is so burnt out, they can’t manage anything. And if we had just been able to get them access to behavioural management a few weeks earlier we could have avoided that admission. But now … they’re headed for long-term care.

James thus felt it necessary to engage in “capacity building for geriatric care across the system”. Sunita (caregiver) conveyed that pharmacists were well-positioned to mitigate iatrogenic harm from conflicting medications when they had a keen clinical acumen (Table 6):

The role of a pharmacist is extremely important because they are the ones who have access to all medications. And a lot of times you go into one doctor and another doctor and they give you different stuff. … The buck stops at the pharmacist because they are supposed to be able to know, “You know what, you can’t take this one [medication] with this one [other medication] because it’s going to do this”.

**Table 6:** Summary of results

Theme	Sub-theme	Quote
1. Shifting culture of care within families, health system resources, and models of care	Cultural shift in caring attitudes to accept the risk of supporting an older adult at home	“You would need a cultural shift to say “I accept the risks of this person being at home. It’s okay if they fall. It’s okay if they’re home alone for a little bit.’ … There’s no way to mitigate the risk. She could fall any time. She could forget to eat anytime. There’s not really a solution … unless you accept a little bit of that risk.”– Maria (palliative care physician)
	The need to better balance the responsibility of the healthcare system with caregiver and community sustainability	“It’s about community development and capacity building in a way that doesn’t make people … feel like, “oh, the government’s trying to get out of paying for things”, dumping everything on the community, everything on the family. … There’s a huge difference between delegating responsibility, building capacity and abdicating responsibility. Unfortunately, we’re in a political climate where there’s a lot of abdication of responsibility or at least pushing things off to the private sector and to individuals.”—Maeve (OT)
	The palliative care model as a starting point for shifting care culture	“If you put that palliative approach on to this chronic senior care, these folks who are in lieu of long term care, I don’t think you can go wrong. … You need a team that’s sort of available on standby who can support when something’s changing. And I don’t think that’s horribly resource intense but I think it is a layer that’s missing.”—Priya (palliative care physician)
	Shifting advanced care planning culture through the lens of family planning and prevention	“When you’re young you never think of aging. … You think of paying the mortgage, getting the family, everything except aging. Then it hits you. … You’re on your own. … Preparing for old age is quite the task.”—Trinh (older adult/caregiver)
2. Shifting the balance of standardized and personalized care: Flexibility as a facilitator to person- and family-centred care	Flexibility in services required to address older adults’ instrumental activities of daily living (iADL)	“1 of my patients who was 94 thought she was fine doing a bath but she needed help making a meal. She said, “Why do I have to get naked to have a sandwich made?” … We have a huge segment of our population … those middle MAPLe’s who’ve got … a little bit of functional decline, perhaps frailty, and they need help with their household chores.”—Fiona (geriatrician)
	Flexibility required to self-identify needs and receive accommodations	“My parents don’t need PSW … but I need help with physio. … It shouldn’t be like pulling teeth. … If you want the seniors to stay out of [long-term care] homes, give them what they need.”—Sunita (caregiver)
	Funding and service flexibility prevents spillover costs: Implications of not having needs met	“[The older adult] was on high dose diuretics and so he was having to go to the washroom way too often. And he was too frail to get up and go. So, he’s wearing incontinence products. But they were too expensive and they’re not covered. So, to prevent himself from having to use so much incontinence products, he cut down on his water pill, which obviously sent him into heart failure, which he was then admitted to hospital and remained in hospital for another 3 or 4 weeks. … So, if we … covered the incontinence products, our system could have saved $250 to $300,000! Like, what!?”—James (geriatrician)
3. Shifting consistency and reliability: Centring older adults and caregivers in care provision	Piecemeal care and the importance of a consistent care-coordinator	“It’s not easy to communicate to someone when we need it, like how to contact, and when to contact. … They make us feel like they’re not remembering us or they just want to give you something and you do it, you complete it. And they’re not … supporting us.”—Ravi (caregiver)
	Consistency and reliability as a facilitator of trust and recognition of needs	“They want to send 3 different PSWs to care for 1 person who has Alzheimer’s. … They’re having a stranger coming to provide personal service for them and tomorrow it’s a different face. … How is that providing any sort of support, compassion, and dignity?”—Lin (caregiver)
	Providers with a high clinical acumen and capacity to treat older adults as central to reliable, preventative care	“the family doc … prescribes Ativan (lorazepam). But it’s well known that Ativan causes falls and cognitive impairment. … So as a geriatrician, I would never use it. … It has to do with the capacity of medical professionals to understand the implications of the drugs that they’re prescribing. And so, yes, this person now has a fall, breaks a hip … and comes into hospital. And I can trace it all back to, ugh, if we had just not done the Ativan.”—James (geriatrician)

## Discussion

Given the gaps in knowledge regarding appropriate home care strategies for older adults in Canada [52], and considering this diverse population’s rapid growth [1], the current study aimed to better understand how to help older adults age at home via co-design and community engagement. The use of the balance of care approach [24, 27–29, 31] and personas [41, 42] was beneficial to anchor discussions and allowed participants to share their lived experiences through grappling with the persona’s circumstances and experiences. Overall, this work demonstrates the social problem and medicalization of aging and broader cultural shift in care (e.g., dominant norms and practices in older adult care) necessary to facilitate aging in place. This cultural shift in care specifically pertained to (1) the shift from being risk-averse to accepting the risk involved in supporting an older adult to age at home, (2) the shift from over-reliance on informal, unpaid caregiving work to strengthened healthcare system resources, (3) the shift from acute or rigid care to more flexible, person-centred care that better accommodates for individual circumstances, (4) the shift from inconsistent services to wraparound, reliable care (e.g., home care providers arriving on time with knowledge of client needs), and (5) the shift from reactive to preventative care (e.g., strengthened clinical acumen for older adults).

Important to participants was proactive care services and better balancing standardization with personalization; this better balance has also been referred to as “individualized standardization” as a way to reconcile these divergent care models [53, p. 349]. De Wit and Schuurmans [54] described a similar paradigm shift needed to “add life to years, not years to life” by (1) building autonomy for older adults, (2) promoting proactive care, and (3) moving from “protocolised to personalized” care [p. 501]. Implementation specialists and policy researchers have also recognized this needed shift “from traditional paradigms” toward “flexible and fit-for-purpose policy, research, and service delivery”; the former is characterized as “grounded in ageism and ableism” with a focus on “standardization and efficiency” while the latter is depicted as a “cataly[st for] sustainable and equitable” services [55, p. 68, 12].

1 way to achieve this better balance between standardized and personalized care, as described by participants in the current study, was allowing older adults and caregivers opportunity to self-identify their needs. Some wanted to self-identify accessibility needs in the home without timely and costly assessments, while others sought to communicate aspects of care with which they struggled most. This is important since engagement in healthcare decision-making is central to supported health and quality of life [56]. Other work has also reported that older adults often lack opportunity to voice their home care service needs resulting, at times, in service discontinuation [57]. Perhaps due to the lack of formal system-level strategies to achieve individualized flexibility, the onus often falls on healthcare providers who then contend with patient needs held up against organizational pressures and professional standards [58].

Formal strategies to achieve this better balance between standardized and personalized care are scarce [59], however, there have been a plethora of recommendations for high quality, person-centred older adult care. For example, Stange et al. [60] proposed 11 domains of quality primary care for older adults, many of which overlapped with what participants in the current study described as lacking in their home care; these included care access, early intervention, strong care-coordination, person-centred focus, knowing the person beyond the patient, and forming trusting clinical relationships. Other research has also found many of these areas to be lacking for culturally and linguistically diverse older adults, particularly surrounding insufficient resources [61], difficult service access [62], lack of knowledge about programs, cultural incompatibility with providers (i.e., lack understanding of diverse older adults’ needs resulting in increased reliance on family members for support) [16, 63], and lacking cultural sensitivity within healthcare services (e.g., food, exchanges with providers) [18, 64–67].

To better prioritize these elements, a relationship-centred model of care has been suggested [60] to centre what is important to older adults [68, 69]. Other work on personalized approaches to older adults’ cardiovascular care posits the importance of goal-directed care (a tenet of patient-centred care); though there is no standard definition or outcome measures, the focus on integrating older adults’ preferences and needs into care, in addition to enhanced communication required to identify shared patient-provider goals, facilitates a shift away from standardization [70]. For culturally and linguistically diverse older adults specifically, shifting away from standardization also includes the need to utilize culturally tailored approaches to promote awareness of available services for specific communities (e.g., promotional material in older adults’ native language, partnering with community organizations to build trust in health services [16, 17]. Dakey et al. [71] similarly recommend the creation of assessment tools that consider culture and language (e.g., assessments offered in many languages, “culturally responsive communication”) in addition to education for healthcare providers on cultural safety [p. 1].

Flexibility was another element of achieving personalization within standard care. Participants described needing conglomerated (rather than broken up) PSW hours; other studies have also noted the necessity of home care scheduling flexibility [55, 72, 73]. Reliability and consistency of the PSW was another issue that participants discussed, making it difficult to build trust, recognize needs, and best respond to older adults. Consistency and reliability were difficult to achieve given the workforce challenges to which participants described (e.g., short staff, inexperienced staff, scheduling difficulties, burn out). Other Canadian studies similarly found difficulty recruiting and retaining home care workers [74] and challenges surrounding scheduling, time constraints, and unpredictable work environments [75]. A proposed strategy to address the growing home care needs of an increasingly diverse population and to retain PSWs thereby thwarting unnecessary long-term care placement, is to increase PSWs’ wages (home care PSWs often have particularly low salaries especially compared to PSWs in other care settings; PSW work is also generally devalued and fulfilled by individuals from equity deserving groups) [76, 77]. Participants in the current study also conveyed the burden of not having a consistent care-coordinator; without assistance, care was often piecemeal, difficult to navigate, and became foisted onto informal caregivers—a finding consistent with other work [78, 79].

Another element of flexibility, described by participants as necessary, was tasks supported, which often did not include iADLs. Other work has emphasized the importance of including iADL supports in home care service packages [23, 25, 27, 29–31, 80]. A particular study aimed to better understand the characteristics of older adults receiving home care in Ontario and Manitoba (Canada), finding that over 90% of their sample (*n* = 174 112) from Resident Assessment Instrument – Home Care data needed iADL support [81]. With scarce resources to engage in iADL support, older adults are sometimes prematurely admitted to long-term care, which ultimately, can become more expensive than community-based care [27]. Since iADL support is important to facilitate aging in place [27, 81–84], these tasks may need to be reconceptualised as key aspects of daily life supported by government funded home care services.

Given the lack of personalization and service flexibility to support ageing in place, participants in this study found it difficult to weave prevention into a reactive “illness cure system”, which often treats aging as a disease. Participants viewed this as “medicalizing a social problem” and discussed the need to reallocate resources upstream to support older adults prior to experiencing impairment. While this is not the first study to discuss aging as a social problem, medicalized in response to lacking resources [85–90], the authors further contextualize this with the need for a better balance between government responsibility and community sustainability. Accessibility and breadth of government funded home care services is thus imperative given the health system’s over-reliance on communities for older adult care [57] and the unaffordability of long-term private home care supports [91, 92]. A particular review on older immigrant’s healthcare experiences in Canada demonstrated that scant government resources prevented older adults from accessing care [63]. These scant resources included a lack of funding aside from pension (which was insufficient to engage in healthy lifestyle habits) [93, 94], the cost of prescriptions not being covered [95, 96] and an “overdependence on informal support networks” for care (e.g., family and friends) [93, 94, 97–100]. When older adults are prevented from accessing care, premature use of institutional care (e.g., long-term care facilities, hospitals) may result [27]. Since healthcare resources are finite, further research is required to better understand how resources may be reallocated toward social care to address the over-medicalization of social issues for diverse older adults. In an often reactive or “illness cure” healthcare system, it is important to shift focus toward preventing avoidable impairment before it occurs.

The authors further contextualize this medicalization of aging as described by participants by drawing attention to the exacerbated health and social challenges faced by diverse older adults. In alignment with other work, participants in the current study conveyed how diverse older adults often experienced financial [101], cultural, and linguistic [63, 102] barriers to care access, which sometimes was attributed to immigration status. In particular, participants pointed to the poor collaboration between immigration policy and healthcare system capacity in Canada, demonstrating how social issues and healthcare challenges are intertwined. The issue was not individuals immigrating to Canada, but rather, a lack of governmental attention to the additional health and social care resources necessary to address the growing population’s needs. Research has demonstrated the particular healthcare challenges that many older immigrant populations often face including lacking cultural integration within the healthcare system [63], obstacles to mental health supports [103] insufficient finances to pay for items like medications [63], and for women in particular, heightened difficulty accessing care [104]. To meet the needs of recent immigrants, change is therefore required within the Canadian healthcare system related to access and awareness, cultural safety, and care planning [105].

In October of 2024, the Government of Canada [106] released a statement on the 2025–2027 Immigration Levels Plan, which will “pause population growth in the short term” with a partial focus on “key labour market sectors such as health” [p. 1]. However, there were no communicated plans to strengthen healthcare system resources to accommodate for those residing in Canada. Nonetheless, in new guidance aims put forth by Canada’s Drug Agency [107], the necessity for services to evolve to better accommodate diverse older adults has been generally acknowledged. Interestingly, a quarter of Canada’s healthcare workers are immigrants with over 40% of new immigrants (2016–2021) engaging in nursing, home care, and residential care facility work [108]. The immigration experiences of healthcare workers in Canada may therefore be an untapped asset; further research is required to better understand strategies to leverage this strength to increase capacity for cultural safety for culturally and linguistically diverse older adults.

The authors recommend looking to other existing models of care to continue strengthening healthcare system resources for diverse aging populations. For example, participants drew attention to the benefits of (A) the palliative care model’s approach to care planning, response to changing needs, and access to providers when needed, and (B) using existing family planning structures to support advanced care planning (e.g., governmental funds, public health education, prevention). Palliative care in Canada is defined as a person- and family-centred approach that “aims to reduce suffering and improve the quality of life for people who are living with life limiting illness” [109, p. 1]. Given its person-and family-centred focus, the palliative model has been recognized as beneficial when integrated early for older adults with cancer (e.g., increased quality of life, better symptom management, increased understanding of the illness faced) [110] and as well suited to older adults with dementia [111–113]. Rosa et al., [114] further posit the benefits of the palliative care model to address “shifting cultural constructs and realities around ageing” [p. 2]. While discussion has been scant regarding the application of family planning models to advanced care planning for older adults, the need for improved advanced care planning support has been clear [115]. The current study extends the need for a model of care for older adults aging in place that draws on the strengths of the palliative care model and mirrors currently available family planning government resources. Further cost-analysis is required to demonstrate the benefit of providing preventative services earlier and over a longer timeframe to avoid what participants believed to be inevitable system-cost implications of lacking support.

To address the need for personalization and flexibility within standard care and to better leverage caregivers’ expertise with regard to what they need to support older adults aging in place, another recommendation is to strengthen self-directed funding programs. Self-directed funding programs allow for autonomous management of some aspects of care and have been implemented across Canada, the United States, Australia, and some countries in Europe [116]. 1 program that currently exists in Ontario is the Family-Managed Home Care Program, which provides funding for home care services or to hire healthcare providers not currently covered by the Ontario Health Insurance Plan. While this program allows for flexibility and would address the need for a consistent healthcare provider, it is limited to children with complex needs or who are home-schooled, adults with brain injuries, and those in “extraordinary circumstances” [117, p.1]. Given the increased control over care and reduced administrative costs found with some self-directed funding programs in Ontario, the authors recommend further work to better understand the implications of this type of program for older adults aging in place and to ultimately extend support to this population [118, 119].

Limitations of this study included that not all culturally and linguistically diverse populations in the Region of Peel were represented, presenting an opportunity for future studies to utilize the study methods (persona development and partnering with community organizations for recruitment) to increase the representation of diverse older adults wanting to age in place. A key consideration to support the recruitment of diverse populations is increasing the representation of diverse populations within the study team who speak the native language of older adult participants. Overall, the co-design and community engagement methods used were effective in answering the research question surrounding the services and resources necessary to support older adults aging in place. These methods prompted open-discussion, and based on what was important to participants, allowed for in-depth exploration of the social problem and medicalization of aging and broader cultural shift necessary to facilitate aging in place. Co-designing personas and related care-packages may be an effective method for other research which aims to better understand communities’ needs and strategies to support various aspects of health and well-being.

## Conclusion

Older adults, caregivers, and healthcare providers in this study described receiving care that was often not person- and family-centered despite this being a purported core healthcare system value in Ontario, Canada. Participants detailed not being given the space to say what they required and generally felt that services fell short of what they needed. A strong message was conveyed that aging is not a disease and that often, inadequate social care leads to the medicalization of aging. This is especially true for immigrant and culturally and linguistically diverse older adult populations who demonstrated the need for person- and family-centred approaches to support aging in place. A cultural shift is therefore needed in medical and social expectations of what it means to care for older adults aging in place.

## Data Availability

All data generated or analysed during this study are included in this published article.

## Abbreviations

MAPLe level: Method for assigning priority level
PSW: Personal support worker
HSW: Home support worker
OT: Occupational therapist
PT: Physical therapist
OTA/PTA: Occupational therapist assistant/physical therapist assistant
